# Antidepressants in Alzheimer’s Disease: A Focus on the Role of Mirtazapine

**DOI:** 10.3390/ph14090930

**Published:** 2021-09-16

**Authors:** Ana Salomé Correia, Nuno Vale

**Affiliations:** 1 OncoPharma Research Group, Center for Health Technology and Services Research (CINTESIS), Rua Doutor Plácido da Costa, 4200-450 Porto, Portugal; anncorr07@gmail.com; 2 Institute of Biomedical Sciences Abel Salazar (ICBAS), University of Porto, Rua de Jorge Viterbo Ferreira 228, 4050-313 Porto, Portugal; 3 Department of Community Medicine, Health Information and Decision (MEDCIDS), Faculty of Medicine, University of Porto, Alameda Professor Hernâni Monteiro, 4200-319 Porto, Portugal

**Keywords:** Alzheimer’s disease, depression, mirtazapine

## Abstract

Mirtazapine belongs to the category of antidepressants clinically used mainly in major depressive disorder but also used in obsessive-compulsive disorders, generalized anxiety, and sleep disturbances. This drug acts mainly by antagonizing the adrenergic α2, and the serotonergic 5-HT2 and 5-HT3 receptors. Neuropsychiatric symptoms, such as depression and agitation, are strongly associated with Alzheimer’s disease, reducing the life quality of these patients. Thus, it is crucial to control depression in Alzheimer’s patients. For this purpose, drugs such as mirtazapine are important in the control of anxiety, agitation, and other depressive symptoms in these patients. Indeed, despite some contradictory studies, evidence supports the role of mirtazapine in this regard. In this review, we will focus on depression in Alzheimer’s disease, highlighting the role of mirtazapine in this context.

## 1. Introduction

Alzheimer’s disease (AD) is a neurodegenerative disease characterized by loss of cognitive ability and loss of ability to perform daily tasks [1]. Depressive behaviors, characterized by apathy, sad mood, anxiety, sleep disorders, and agitation, are often present in AD patients [2]. Thus, the management of these conditions is essential for AD patients, aiming for an improvement in their quality of life and clinical outcome. There are several therapeutic modalities in this context including pharmacotherapy and non-pharmacological approaches, such as psychotherapy and physical exercise, if possible [3]. However, depression in AD is extremely inter-variable, appearing as a risk factor, early signal, or symptom of the disease. Therefore, it is extremely difficult to manage. Furthermore, the symptoms of major depressive disorder (MDD) and dementia can often overlap, leading to increased difficulty in the diagnosis [4]. Thus, there are no universally accepted treatments, and they must be suitable for each patient, requiring careful examination. However, in general, selective serotonin reuptake inhibitor drugs (SSRIs) are the best tolerated in this context, due to their efficacy with less associated side effects [2]. Even so, other drugs that have a dual mechanism of action are, also, often prescribed to these patients, such as mirtazapine [5].

Thus, this article aims to provide an overview of depression in AD, highlighting pharmacological evidence, with special emphasis on mirtazapine, a noradrenergic and specific serotonergic antidepressant with some evidence in this disease, although sometimes contradictory and not very clear. Antidepressant treatment in AD is a very important research topic, with some consensual gaps. The study of this issue is crucial, since the treatment of neuropsychiatric disorders in AD is essential to the well-being of the patients, and it may even lead to cognitive improvements by acting on beta-amyloid (Aβ) pathology and tau hyperphosphorylation, characteristic hallmarks of AD, despite the need of more studies to clarify these mechanisms [4].

## 2. Alzheimer’s Disease and Depression

### 2.1. Alzheimer’s Disease: A Brief Characterization 

AD is the most common cause of dementia in the world, with most cases occurring in individuals over 65 years old [6]. It is extremely important to study this pathology, as studies indicate that the incidence of dementia will triple worldwide in 2050 [7]. Today, about 50 million people worldwide suffer from dementia (AD in about 70% of cases) [8].

**Figure 1:**
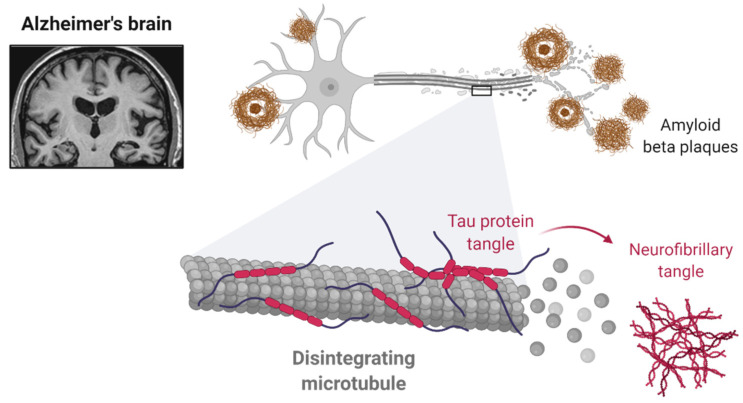
The hallmarks of AD are the presence of a Aβ plaques and neurofibrillary tangles. Reprinted from “Alzheimer’s Brain (Disintegrating Microtubule)”, by BioRender.com (2021). Retrieved from [18]. Available online: https://app.biorender.com/biorender-templates (accessed on 16 August 2021).

AD is characterized by three typical stages: preclinical, pre-dementia phase and, finally, dementia. The preclinical (or clinically asymptomatic) and pre-dementia phases may last for several years, preceding dementia, where there is present a severe cognitive impairment. In the preclinical phase, the patients are asymptomatic. However, when the production of the Aβ peptide reaches a threshold, the critical accumulation of Aβ peptide leads to a stage where the patient starts having some symptoms, namely subtle memory loss and, sometimes, neuropsychiatric symptoms, frequently overlapping with the diagnosis of other neuropsychiatric disorders, such as MDD [9,10]. An accurate diagnosis of behavioral symptoms is particularly important in the early phases of AD. Thus, after the preclinical stage, AD is characterized by symptoms such as progressive memory loss, aphasia, apraxia, agnosia and progressive difficulty in living a normal lifestyle [1]. In addition to the cognitive symptoms mentioned above, it is important to refer the importance of neuropsychiatric symptoms, which occur in practically all patients with AD. These symptoms include depression, apathy, agitation (physical and verbal) and circadian rhythm disturbances. As the disease progresses, hallucinations and aggressive behavior are also frequently observed [11]. Regarding the pathophysiology, this disease is characterized by the presence of neurofibrillary tangles and the accumulation of Aβ protein in the brain (Figure 1) [12]. Neurofibrillary tangles are accumulations of hyperphosphorylated tau protein in several brain areas that lead to the loss of cytoskeletal proteins (such as microtubules) and other proteins essential to the normal cell function, such as tubulin-associated proteins [13]. In turn, the extracellular accumulation of Aβ protein forms the so-called senile plaques, which play a preponderant role in neural function, leading to neurotoxicity and consequent dysfunction of several brain areas, such as the hippocampus, resulting in progressive cognitive decline [1,14]. Furthermore, other factors such as nervous tissue inflammation, oxidative stress and dysfunction of the cholinergic system are also important in the pathophysiology of AD [15,16,17].

### 2.2. Depression in Alzheimer’s Disease

Depression is one of the most prevalent psychiatric diseases in the world, being a highly debilitating illness [19,20]. It is a heterogeneous disease, characterized by anhedonia and a sad mood, which can lead to death by suicide and, also, impairment of cognitive functions, such as memory and learning [21,22]. However, the full comprehension of this disease is a major challenge for neurosciences since it has a complex and not fully understood pathophysiology. Indeed, it involves several systems, from the immune system to the neuroendocrine system, in addition to several molecular components, such as neurotransmitters [23]. Despite all the complexity and lack of knowledge associated with depression, there are hypotheses proposed in recent decades to explain its pathophysiology: the neurotrophic, neurogenic and monoamine hypotheses [24]. Focusing on the most accepted hypothesis, the monoamine hypothesis, this theory is based on the central premise that the concentration of serotonin (5-HT) in synaptic transmission is low during depressive episodes. Nevertheless, there is also a recognized role of other monoamines in this condition, namely NE and dopamine [25,26]. Thus, 5-HT deficiency has been widely associated with the etiology of depression.

Depression is highly associated with AD, with about 30% prevalence in Alzheimer’s patients [27]. It is a behavioral disease that can accelerate the patient’s cognitive decline. Thus, management and understanding of this condition is crucial [28]. Overlapping symptoms between AD and depression are usually present, such as sleep disturbances, anxiety, apathy, and hyperactivity [29]. Therefore, the study and recognition of neuropsychiatric symptoms associated with AD is extremely important and clinically relevant, aiming to improve the patient’s life quality. Indeed, depression in elderly patients can also be considered a sign or a risk factor for the development of dementia, requiring a careful assessment of the patient condition, since the diagnosis of these diseases is very challenging. In early AD, depression symptoms generally translate into a sadder mood and depressive thoughts, while in more advanced states, problems with agitation, aggression and circadian rhythm alterations are more evident [5]. Another important finding is the fact that about a third of adults who have depression are also diagnosed with mild cognitive impairment (MCI), which precedes severe forms of AD [30]. Some studies even show that the presence of depression in individuals with MCI favors the subsequent onset of AD [31]. Also, family and personal history of depression [32,33], positivity for the *apolipoprotein susceptibility gene E4* (ApoE4) [34] and the use of certain medications such as corticosteroids or benzodiazepines are examples of risk factors that are associated with the presence of depression in AD patients [35].

Patients with AD and concomitant depression tend to have more severe pathophysiology (for example, more Aβ deposits in the brain) and more problems in the serotonergic system [36]. Nevertheless, changes in the levels of 5-HT and other neurotransmitters, such as NE, are related to depression in AD [4]. Particularly, in vivo studies in mice models of AD have shown that decreasing NE levels lead to increased levels of neuroinflammation and Aβ peptide deposits [37]. Regarding the role of 5-HT, studies show that decreased levels of this neurotransmitter lead to increased production of Aβ peptide, by modifying the cleavage capacity of amyloid precursor protein (APP) [38].

The over-activation of the HPA axis observed in depression and the exaggerated secretion of glucocorticoids leads to reductions in hippocampal volume, contributing also to neurodegeneration and dementia [39]. Indeed, increased levels of expression of APP induced by glucocorticoids have been demonstrated in several studies [40].

Depression is also connected to a decrease in neurotrophic factors, such as brain-derived neurotrophic factor (BDNF) [41]. This growth factor is very important in several processes, such as neurodegenerative processes, interacting with the serotonergic system by enhancing the survival of serotonergic neurons [42]. Indeed, BDNF gene polymorphisms are associated with reduced volumes in the hippocampus, frequently observed in depressed patients and contributing to neurodegeneration, typically present in AD patients [38].

Exacerbated neuroinflammation, reflected in the over-activation of pro-inflammatory pathways (for example, increased C-reactive protein secretion), is also a factor that leads to the neurodegeneration phenomena [43,44], as well as chronic microglial activation, observed in both depression and AD. Indeed, genes for immune receptors are abundantly expressed in these cells, such as triggering receptor expressed on myeloid cells 2 (TREM2), known to participate in the development of AD [4,45].

Highlighting the relationship between AD and MDD, a study has also shown that patients with Aβ brain deposits are about four times more likely to develop depression and other neuropsychiatric symptoms [46]. Another recent longitudinal study also concluded that higher levels of cortical amyloid correlate with worse depressive episodes, suggesting that depression is a potential target in AD treatment, aiming to delay fast cognitive decline [47].

**Figure 2:**
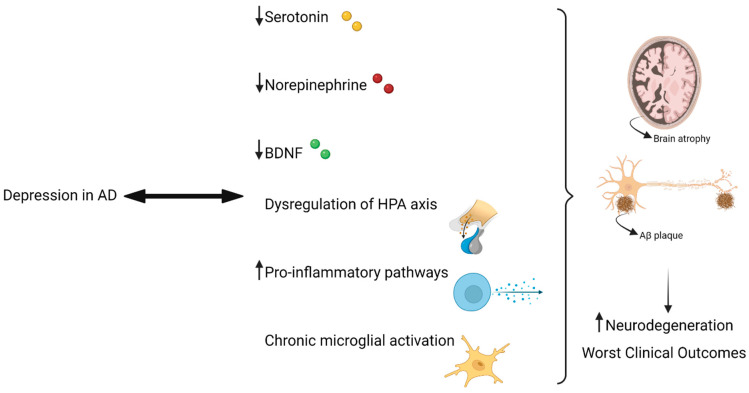
Findings such as decreased levels of 5-HT, NE, and BDNF, as well as dysregulation of the HPA axis and pro-inflammatory pathways, are associated with depression, contributing to the increase in neurodegeneration phenomena present in AD, such as the presence of Aβ plaques and brain atrophy. Adapted with permission from ref. [48]. Copyright 2019 Frontiers Media S.A. Created with BioRender.com [49]. Available online: https://biorender.com/ (accessed on 16 August 2021).

In sum, depression in AD can appear in several ways: as a risk factor, an early sign of neurodegenerative changes, or also as a resultant symptom of the disease. Either way, depression leads to several molecular mechanisms that can aggravate the condition of AD patients, or even lead to the progression of the disease from milder to more severe levels [4]. Figure 2 summarizes the relationship between depression and AD.

### 2.3. Antidepressants in Alzheimer’s Disease

There is a lot of evidence, sometimes not totally clear, about the use of antidepressants in AD [29]. However, it is known that these drugs have neuroprotective effects in the context of depression. Indeed, as a result of increased 5-HT levels, there is evidence that cognitive activity and long-term memory may be improved [50]. Nevertheless, the impact of antidepressant therapy on AD remains unclear, despite a known relationship between both pathologies [4]. Notably, antidepressants are associated with a reduced risk of developing AD, while there is no clear evidence that these drugs act on the progression of AD. Nevertheless, by acting on inflammatory pathways and the balance of neurotransmitters, antidepressants can then delay the onset of AD [51,52]. Moreover, a great variety of antidepressant drugs have the potential to manage depression in AD. However, the low risk of side effects and drug interactions makes SSRIs the class of drugs most commonly prescribed in this context [2].

Studies with SSRIs have shown benefits in the use of this class of drugs in the context of depression in AD [4]. A retrospective study that evaluated the relationship between the risk of AD and the use of SSRIs in depressed patients, concluded that individuals who took these drugs chronically had a lower risk of developing AD, compared to patients who only took these drugs in a short period [53]. Other studies also demonstrate that individuals with AD and MCI improved their cognitive, memory capacity and depressive symptoms by taking fluoxetine and sertraline, improving the quality of life of these patients [54,55]. In another study, the long-term use (over periods longer than four years) of SSRIs in patients with MCI was associated with slower progression to AD [56]. Other studies in animal models demonstrated that this class of drugs led to a reduction in Aβ plaques which, in turn, reduced the cognitive decline [57]. Modulation of several important factors in neuroinflammation processes (such as tumor necrosis factor α and IL-6) has also been associated with SSRIs [58]. In mouse models of AD, citalopram (an example of SSRI) reduced Aβ peptide in brain interstitial fluid. Indeed, this drug decreased the growth of already existing plaques and reduced de novo production of plaques [59]. Another example of SSRI is fluoxetine. In preclinical studies, in animal models of AD, this drug increased the size of the hippocampus and dentate gyrus and promoted the expression of proteins related to the activation of the CREB/BDNF signaling pathways [60]. Furthermore, it reduced the amount of soluble Aβ peptide in both cerebrospinal fluid, brain tissue and blood. In this study, it was also observed that this drug led to improvements in the animals’ memory (specially spatial memory), at the behavioral and cognitive levels. Moreover, it also prevented the loss of proteins involved in the synapse (such as synaptophysin and microtubule associated protein 2) and to the inhibition of the phosphorylation of the APP, eventually reducing the capacity to produce Aβ peptide [61]. In another study, it was also observed that astrocytes present in an APP/presenilin 1 mouse model (characteristic model of AD disease) produced large amounts of soluble Aβ peptide, a process that was inhibited by fluoxetine, that activated 5-HT2 serotonergic receptors. In this study, fluoxetine also promoted neuroprotection against the damage induced by this type of glial cells [62]. Also with this drug, another study in cell lines showed that fluoxetine decreased the toxicity induced by the Aβ peptide, depending on the paracrine signaling mediated by transforming-growth-factor-β1 (TGF-β1), an anti-inflammatory cytokine, which promotes neuroprotection in the context of neurodegeneration observed in AD [63]. Escitalopram is another drug belonging to the class of SSRIs. Studies in rat hippocampal neurons treated with Aβ42 peptide revealed that this drug reduced tau hyperphosphorylation, a feature present in AD. This reduction in the hyperphosphorylation of tau was due to the action of this drug on the 5-HT1A serotonergic receptor, namely via Akt/GSK-3β pathways [64]. Regarding paroxetine, also belonging to the class of SSRIs, studies in animal models of AD concluded that this drug improved behavioral aspects in animals, as well as reduced Aβ peptide levels and problems associated with tau protein [65]. In addition, fluvoxamine (other SSRI) significantly improved memory function in an AD model of mice, by inhibiting γ-secretase activity and, consequently, reduced Aβ peptide generation [66]. Another study focusing on the efficacy of several SSRIs (fluvoxamine, fluoxetine, paroxetine, sertraline, and escitalopram) in AD, evaluated these drugs on Aβ42 aggregation and generation of fibrils, concluding that this class of drugs (highlighting fluoxetine and paroxetine) can inhibit Aβ42 aggregation and reduce fibrillogenesis [67].

In addition to SSRIs, other antidepressants have also shown evidence in the context of AD. An example is mirtazapine, which we will only focus on Section 3. Another example is trazodone, an atypical antidepressant. In a recent study, this drug retarded cognitive decline, as well as improved sleep disorders in AD patients [68]. Amoxapine, a tricyclic antidepressant (TCA), is another example of a potential drug in the context of AD associated depression. Studies have shown that this drug can be beneficial in AD disease, reducing the production of Aβ peptide and improving cognitive function, by acting on the 5HT6 serotonergic receptor [69]. In studies on mice neurons, amitriptyline (also a TCA drug) reduced neuronal death after exposure to the Aβ42 peptide. Furthermore, this drug increased the expression of the genes activating transcription factor 3 and heme oxygenase, which are important in terms of neuroprotection [70]. With desipramine, another antidepressant that belongs to the class of TCAs, in mice, it was observed that this drug improved the depressive behavior, as well as the working memory in these animals, reversing the effects caused by the Aβ42 peptide and promoting neuroprotection, mainly through the up-regulation of CREB phosphorylation in the hippocampus of these animals [71]. Imipramine (another TCA), in AD mouse models, also led to improvements in cognition and memory, as well as reduced Aβ peptide accumulation in these animals. This mechanism is based on the inhibition of TNF-α, a pro-inflammatory cytokines with relevant roles in the neuroinflammation observed in AD patients [72].

In studies with cell lines, moclobemide, characterized as a monoamine oxidase (MAO) inhibitor, increased the proliferation of hippocampal progenitor cells, as well as BDNF levels [73], having potential for AD. Another MAO inhibitor is tranylcypromine (TCP). In a study with cortical neurons, this drug greatly reduced neuronal death induced by the Aβ42 peptide. In this study, derivatives of this drug (TCP butyramide and TCP acetamide) also prevented neurodegeneration caused by the Aβ peptide, representing another possible therapy applicable to AD [74]. Other compounds with antidepressant properties, such as NMDA receptor antagonists, have also shown efficacy in the context of AD. An example is the drug ketamine, which, by activating the mammalian target for rapamycin (mTOR) pathway, exerts antidepressant effects in AD, particularly at the behavioral level [75]. Dysregulation of this pathway has been observed in postmortem brains of Alzheimer’s patients [76]. Table 1 summarizes the drugs mentioned in this text, highlighting some evidence in AD, namely in the context of depression associated with this disease.

## 3. Mirtazapine

### 3.1. Characterization and Clinical Indications

Mirtazapine is an antidepressant drug that was first synthesized in 1987 in the Netherlands [82]. This drug acts on the serotonergic and noradrenergic system, classified as a tetracyclic antidepressant, more particularly noradrenergic and specific serotonergic antidepressant (NaSSA) [83]. Mirtazapine is primarily indicated for the treatment of MDD [84], having other indications as well [82], described in Table 2. Sedative, antiemetic, appetite-stimulating effects (hyperphagia) and the presence of nightmares are commonly associated with this drug [83,85,86]. Clinically, mirtazapine is prescribed for MDD when first-line therapies (such as SSRIs) fail, proving to be effective in various stages of this disease, as well as in associated symptoms such as insomnia and generalized agitation [82]. Additionally, the combination of mirtazapine with SSRIs such as paroxetine and fluoxetine, or with serotonin-norepinephrine reuptake inhibitors (SSNRIs) such as venlafaxine is frequent, being, in some cases, recommended for the treatment of MDD [87,88,89].

Compared to SSRIs such as sertraline, this drug has also a faster action, an advantage over these drugs [90]. Furthermore, this drug has practically no anticholinergic or 5-HT -related side effects, having a favorable safety profile [82]. However, as referred above, there are some side effects associated with mirtazapine, being weight gain and somnolence the most common [91]. Nevertheless, some studies indicate that for the treatment of depression, there is no established difference between the efficacy of mirtazapine and other antidepressant drugs, but there is a greater possibility of remission after treatment with mirtazapine [92]. Other studies also point out that mirtazapine is one of the most effective antidepressants in the treatment of MDD, when a total of 21 different antidepressants were compared [93]. In another more recent study, which analyzed several SSRIs (fluoxetine, paroxetine, sertraline, citalopram, and escitalopram), venlafaxine, and mirtazapine in the treatment of MDD, it was found that mirtazapine was effective and well-tolerated in the treatment of this disease at lower doses, reducing the chance of significative side effects. In this study, when specifically compared with venlafaxine, the efficacy of mirtazapine increased up to a dose of 30 mg, whereas with venlafaxine, the dose-efficacy relationship increased with doses up to 75–150 mg [94].

Mirtazapine is still widely prescribed for the treatment of MDD, requiring a personalized assessment of the patient, since depression is a highly complex and inter-variable disease.

### 3.2. Mechanism of Action and Pharmacokinetics of Mirtazapine

**Figure 3:**
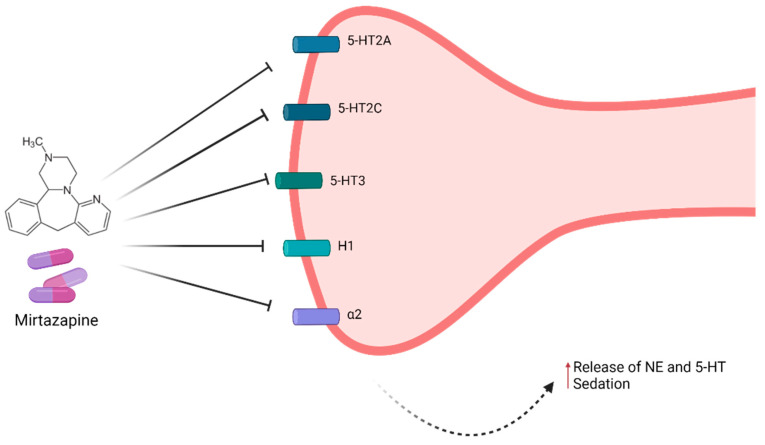
Summary of the mechanism of action of mirtazapine. This drug is an antagonist of 5-HT2A, 5-HT2C, 5-HT3, H1, and α2 receptors, resulting in antidepressant and sedative effects [107]. Image for illustrative purposes only. Created with BioRender.com [49]. Available online: https://biorender.com/ (accessed on 16 August 2021).

Mirtazapine acts as an antagonist of 5-HT receptors: 5-HT2 (5-HT2A and 5-HT2C) and 5-HT3, resulting in the stimulation of 5-HT1A receptors and, consequently, enhanced serotonergic transmission, a factor that is crucial in the antidepressant action of this drug [107,108]. Regarding the noradrenergic transmission, this drug blocks central α2 hetero and auto-receptors, promoting noradrenergic signaling, which consequently leads to an increase in the release of both 5-HT and norepinephrine (NE) [108]. This activation in the sympathetic nervous system is also extremely important in the antidepressant activity of mirtazapine [83]. In addition to these targets, mirtazapine has a sedative action, a result of the antagonistic activity on histamine receptors (H1) [83,109]. Moreover, this drug also possesses an affinity for muscarinic cholinergic receptors and peripheral α2 receptors, albeit to a low degree, as well as a practically negligible affinity for dopamine receptors. Furthermore, mirtazapine has no activity in the reuptake of neurotransmitters, namely 5-HT, dopamine, or NE [83,86,107]. Another important effect of mirtazapine is a high reduction in cortisol levels, as well as the control of the hypothalamic-pituitary-adrenal axis (HPA) [110]. In Figure 3, the mechanism of action of mirtazapine is summarized.

Pharmacokinetically, this drug is absorbed in the gastrointestinal tract and metabolized in the liver via the cytochrome P450 enzymes (CYP1A2, CYP2D6, and CYP3A4), and its metabolites are eliminated in the urine and, to a lesser extent, in the feces. Additionally, it is important to note that this drug has a half-life of 20–40 h [111].

### 3.3. Mirtazapine in Alzheimer’s Disease

Mirtazapine is a drug prescribed in the context of depression and agitation associated with AD, with several and sometimes contradictory studies [112].

Indeed, an older study reported that patients with AD and associated depression, after taking mirtazapine, greatly improved their depressive symptoms (lack of appetite, anxiety, insomnia and anhedonia). However, symptoms associated with AD remained (such as memory loss), showing potential for further study of this drug in the context of depression in AD [113]. Another prospective study showed that in AD patients whose agitation and lack of appetite was exacerbated, the treatment with mirtazapine led to significant improvements in these symptoms, with no relevant side effects, supporting the use of this drug in AD patients with this type of associated symptoms [114]. However, other study evaluated the efficacy and safety of two widely prescribed antidepressants in the context of depression associated with dementia, mirtazapine and sertraline, versus the use of placebo. This study concluded that there were no significant differences in the level of efficacy between patients who took mirtazapine, sertraline, and placebo. However, in patients who were treated with antidepressants, the risk of occurrence of adverse effects increased compared to placebo [115]. Thus, with this study, it was concluded that the use of these drugs in AD should be studied in greater depth, as they showed no benefit, possibly due to the heterogeneity of depressive symptoms in patients with dementia [115,116]. Based on the results of this study, the effectiveness of these same drugs was again evaluated, but in depression subgroups, in patients with dementia (severe, psychological, affective, and somatic subgroup). Only in the “psychological” subgroup, where symptoms such as anxiety and pessimism were present, but without sleep disturbances, mirtazapine proved to be effective, compared to sertraline and placebo [116]. Another study in AD patients with sleep disorders evaluated the effectiveness of mirtazapine compared to placebo, regarding the treatment of these sleep problems. Once again, mirtazapine was not effective in the treatment of sleep disorders (regarding the duration and quality of nighttime sleep), increasing the daytime sleepiness [117]. Moreover, another recent investigation (based on the collection of opinions from experts in the field) demonstrated that in patients with AD and depression, one of the antidepressants of choice is mirtazapine, characterized by having a dual mechanism of action and by leading to improvements in cognitive function/depressive episodes [5]. Furthermore, a systematic review and network meta-analysis concluded that sertraline and mirtazapine improved depressive symptoms, with no differences in cognitive function. Thus, these drugs are therapeutic options that can be considered in the context of depression in AD [118]. Another very recent study in rat hippocampal neurons, found that mirtazapine was able to reverse the atrophy of neurites, caused by exposure to the Aβ peptide. Also, in this study, mirtazapine promoted the trafficking of Golgi vesicles in dendrites, previously inhibited by exposure to Aβ peptide. This vesicular traffic is essential for the maintenance of neuronal polarity and, consequently, for good neuronal functioning, typically dysregulated in AD. These findings, although preclinical, support the use of mirtazapine as an antidepressant with considerable potential in AD [119].

It is important to refer that mirtazapine can be prescribed alone or in combination with other drugs. Frequently, it is important to act in several signaling pathways that are involved in a disease, increasing the efficacy of the treatment. The drugs donezepil, galantamine, rivastigmine (cholinesterase inhibitors) and memantine (NMDA receptor antagonist) are the most prescribed for AD [120], and their combination with mirtazapine does not present lack safety or efficacy issues. This leads to the possibility of mirtazapine to be administered in a combination therapy for AD patients, eventually leading to better outcomes by targeting different signaling pathways [114,121]. Additionally, the combination of antipsychotics (such as risperidone and quetiapine) and mirtazapine may be also observed in AD therapeutic regimens [122,123].

Thus, although several contradictory and unclear information, mirtazapine is still widely prescribed for depression in AD patients [5]. However, a careful and individualized examination of the patient is needed, as well as more studies about this drug in the context of AD-associated depression.

## 4. Conclusions

Depression in AD is a very frequent complication in this disease, being extremely important to be managed, as it can accelerate cognitive decline and significantly reduce the patient’s quality of life. It is crucial to study the mechanisms behind this condition, so that the treatment may be as effective as possible, with the fewest side effects. Thus, it is important to continue to investigate the effect of the many antidepressants for this condition, since there is plenty of evidence about their benefits, although there are some contradictions in the literature. SSRIs are the most prescribed drugs in this context, but it is important to consider the high inter-variability, being necessary to extend the study to other classes of antidepressants. In this context, mirtazapine also appears as a highly prescribed drug, despite some unclear evidence. Nevertheless, this drug has proved to be effective in most of the cases, especially in the context of the agitation observed in these patients. More investigation about this topic of studies is absolutely relevant and needed.

## Figures and Tables

**Table 1:** Summary of the antidepressants addressed in this review, highlighting some evidence in AD and the general mechanism of action, by drug class.

Drug	Drug Class	General Mechanism of Action	Examples of Evidence in AD
Fluoxetine Citalopram Escitalopram Paroxetine Fluvoxamine Sertraline	Selective serotonin reuptake inhibitors	This class of drugs inhibits the serotonin transporter (SERT) at the neurons, thereby enhancing the concentration of 5-HT in the synaptic cleft [77]	Increase in the hippocampus size, reduction of the amount of soluble Aβ peptide, improvements in memory, cognition, behavior, life quality, reduction of tau hyperphosphorylation, modulation of neuroinflammation
Desipramine Imipramine Amoxapine Amitriptyline	Tricyclic antidepressants	This class of drugs inhibits the serotonin transporter (SERT) and norepinephrine transporter (NET), enhancing the concentration of serotonin and norepinephrine in the synaptic cleft. Additionally, they are antagonists of α1 and α2, muscarinic, and H1 receptors [78]	Reduction in the production and accumulation of Aβ peptide, reduction in neuronal death and neuroinflammation, improvement in cognitive function, neuroprotection, cognition, and memory
Moclobemide Tranylcypromine	Monoamine oxidase inhibitors	This class of drugs inhibits monoamine oxidase enzyme, an enzyme responsible for the breakdown of several neurotransmitters such as 5-HT, NE and dopamine [79]	Increase in the proliferation of hippocampal progenitor cells, BDNF levels and reduction of neuronal death
Trazodone	Atypical antidepressant: serotonin-antagonist-and-reuptake-inhibitor [80]	This drug inhibits SERT and 5-HT2 receptors [80]	Delay of cognitive decline, improvement of insomnia
Ketamine	NMDA receptor antagonist	This drug acts mainly by antagonizing NMDA and glutamate receptors [81]	Behavioral improvement

**Table 2:** Indications of mirtazapine in neuropsychiatric disorders that do not include MDD.

Indication	Description
Insomnia	Decreases REM sleep, improves the quality of sleep and sleep continuity [95]
Panic disorder	Decreases agitation and panic attacks; Can be a fast and effective alternative to SSRIs [96,97]
Post-traumatic stress disorder (PTSD)	Effective and well-tolerated [98]; Effective in PTSD related to combat [99]
Obsessive-compulsive disorder (OCD)	Effective with continuous treatment [100]; Fast action, more effective and fewer side effects when added to citalopram [101]
Anxiety disorders	Effective and earlier-onset action (vs. paroxetine) [102]; Effective in reducing generalized anxiety [103]; Lack of efficiency in social anxiety disorder (vs. placebo) [104]
Migraine	Prevents migraine initiation and treats this condition [105]; However, side effects of mirtazapine’s use include headaches [106]. Thus, there is a lack of consistent evidence

## Data Availability

Not applicable.
